# Cancer Risk in Patients with Gaucher Disease Using Real-World Data

**DOI:** 10.3390/jcm12247707

**Published:** 2023-12-15

**Authors:** Shoshana Revel-Vilk, Ari Zimran, Majdolen Istaiti, Liat Azani, Varda Shalev, Gabriel Chodick, Orly Manor, Ora Paltiel

**Affiliations:** 1 Gaucher Unit, Shaare Zedek Medical Center, Jerusalem 9103102, Israel; azimran@gmail.com (A.Z.); joleenist@szmc.org.il (M.I.); 2 Faculty of Medicine, The Hebrew University of Jerusalem, Ein Kerem, Jerusalem 9112102, Israel; orap@hadassah.org.il; 3 Braun School of Public Health and Community Medicine, Hebrew University, Jerusalem 9112102, Israel; orlyma@ekmd.huji.ac.il; 4 MaccabiTech, Maccabi Healthcare Services, Tel Aviv 6772168, Israel; 5 School of Medicine, Tel Aviv University, Tel Aviv 6997801, Israelchodick@tauex.tau.ac.il (G.C.)

**Keywords:** Gaucher disease, cancer, screening bias, surveillance bias, real-life data

## Abstract

The association between GD and cancer has been uncertain due to ascertainment bias in previously published studies. We analyzed cancer incidence using the Maccabi Healthcare Service (MHS) electronic health records among 264 patients with GD compared to 3440 matched controls. We ascertained cancers diagnosed before and after the index date (i.e., the first documentation of GD in cases and the corresponding date for controls). Before the index date, cancers were diagnosed in 18 individuals, with 11 (4.2%) in the GD group and 7 (0.2%) in the control group. After the index date, cancers were diagnosed in 57 individuals, with 20 (7.9%) in the GD group and 37 (1.1%) in the control group, with a median follow-up of almost 13 years in both groups. The most common cancers diagnosed in GD were non-melanoma skin cancer (NMSC) and hematological malignancies, with a clustering of diagnoses around the time of GD diagnosis. The incidence of cancers (excluding MNSC) was 4.1 (95% CI 2.2–7.1) and 0.7 (95% CI 0.4–0.9) per 1000 patient-years in the GD and control groups, respectively, with an incidence rate ratio of 6.37 (95% CI 3–12.7). Patients with GD underwent more cancer screening tests than their counterparts in the control group. While our study revealed an increased occurrence of cancers in patients with GD, this finding might be partly attributed to the more rigorous surveillance procedures employed in this patient population.

## 1. Introduction

Gaucher disease (GD), a rare autosomal recessive disorder, is characterized by a deficiency of the lysosomal enzyme β-glucocerebrosidase, leading to the accumulation of glucosylceramide in macrophages throughout the body. Accordingly, the primary disease features are hepatosplenomegaly, hypersplenism, anemia, thrombocytopenia, and bone involvement [1]. There are three types of GD, with a combined estimated occurrence ranging from 0.45 to 25.0 per 100,000 live births. However, among individuals with Ashkenazi Jewish heritage, type 1 GD is considerably more prevalent, with an estimated occurrence of 1 in 850 live births [2]. In the non-Ashkenazi Jewish population, GD is far more severe, there are more cases with neuronopathic forms, and it may be life-threatening [3].

One of the most challenging aspects and sources of controversy surrounding GD is the issue of associated diseases, both GD-related and GD-unrelated. While Parkinson’s disease and cholelithiasis are clear examples of GD-related comorbidities, there is uncertainty regarding the association between GD and cancer, a topic that raises great concern for patients and their families. There are numerous reports of cancers in patients with GD, mostly from single-center studies [4,5,6], with conflicting results as to whether patients with GD have an increased risk of malignancy [7]. Several studies have reported an increased risk of multiple myeloma, occurring 6–50 times more often than expected in individuals with GD [4,5,8]. The risk of other hematologic malignancies and overall cancer risk was also reported to be elevated [5]. Others did not find an increased risk of overall cancer compared to expected rates [6,8,9].

A recent study conducted by the International Collaborative Gaucher Group (ICGG) GD Registry, with 2123 adult patients, showed an increased risk of hematologic, liver, renal, melanoma, and breast malignancies [10]. Although performed using a relatively large multicenter cohort, this study had several limitations. First, the data were based on a voluntary, observational registry, with a risk of selection bias. Second, the overall risk for cancer and each type of malignancy in patients with GD was compared to the general US population using the Surveillance, Epidemiology, and End Results database, whereas the population with GD is over-represented by patients of Ashkenazi Jewish heritage. Third, other non-GD-related risk factors for malignancy were not studied. Uncertainty remains regarding the true risk of cancer among patients with GD and the contribution of surveillance bias to the seemingly heightened risk.

The aim of the current study was to evaluate the risk of cancer in patients pre- and post-diagnosis of GD compared to a group of matched controls in a population-based comprehensive clinical database.

## 2. Methods

### 2.1. Study Cohort

This study utilized electronic health records from the Maccabi Healthcare Service (MHS), Israel’s second-largest health maintenance organization. The MHS has computerized clinical records for over 20 years, seamlessly integrated with an automated central laboratory, digitized imaging, and pharmacy purchase data.

The study cohort included individuals with a confirmed diagnosis of GD in the MHS database and 13 randomly selected controls per GD case, matched for year of birth, sex, and socioeconomic status (per MHS data). The confirmation of GD diagnosis was previously described [11]. In brief, patients with an MHS diagnosis code for GD were included if they had evidence of GD-specific treatment authorization or medical notes indicating the diagnosis of GD. Five individuals (one with GD and four controls) with no follow-up data were eventually excluded from the analysis. This resulted in a final cohort of 264 patients with GD and 3440 controls (Table 1). The index date for GD diagnosis was the first documentation of GD in the MHS database. For the controls, the index date used was the index date for the GD diagnosis of the matched patient. Around 25% of patients and controls were older than 60 at the end of the follow-up, with no age difference between the groups.

### 2.2. Data Extraction

Data on cancer diagnoses were extracted from the MHS cancer register, which captures all types of cancer and was updated until 31 January 2021. Additional data extracted included demographic information, ICD-9 codes for a family history of cancer, smoking history and history of splenectomy, use of GD-specific medication, and MHS codes for the performance of colonoscopy, mammography, occult blood in stool, Papanicolaou (PAP) smear, prostate-specific antigen (PSA), and skin screen. Tests performed following a diagnosis of cancer were not included.

The study protocol received approval from the MHS Institutional Review Board (0013-21-ASMC). This study did not require patient consent since de-identified data were utilized, waiving the need for individual patient consent.

### 2.3. Statistical Analyses

Descriptive statistics are presented as frequencies, proportions, mean and standard deviation (SD), or median and range, as appropriate. Proportions or medians were compared across groups using the Chi-square test for categorical variables and the Wilcoxon test for non-parametric continuous variables.

To compare patients with GD to controls, the analysis was performed separately for patients diagnosed with cancer before and after the index date.

Cancer-free survival after the index date for patients with GD and controls was assessed using Kaplan–Meier methods, and comparisons were made using the log-rank test. Cancer incidence rates among patients with GD and controls were calculated and compared using the incidence rate ratio with 95% CI. Cases diagnosed with cancer before the index date were excluded from these analyses. The probability of cancer was assessed for the first presenting cancer in patients diagnosed with multiple cancers. Analyses were performed with and without including patients developing non-melanoma skin cancer (NMSC).

The assessment of risk factors for cancer in patients with GD was conducted for patients with GD who were older than 20 years with a follow-up of at least one year. The analysis included the following variables: age, sex, socioeconomic status, history of splenectomy, and treatment with GD-specific medication. Patients were considered treated if the GD-specific medication was prescribed at least six months before the cancer diagnosis. Univariate and multivariate Cox proportional hazard analysis was used to study variables associated with cancer risk in patients with GD.

Analyses were conducted using R version 2022.07.01.

## 3. Results

### 3.1. Cancer Incidence before Index Date in Patients with GD Compared to Controls

Cancer was diagnosed in 18 individuals before the index date: 11 (4.2%, 95% CI 2.1–7.3%) among patients with GD and 7 (0.2%, 95% CI 0.08–0.42%) among controls (Table 2). The main types of cancers in patients with GD were NMSC and hematological cancers.

The median follow-up from the first record in the MHS database to the index date was slightly higher in patients with GD, 6.4 years, compared to controls, 5.3 years (*p* = 0.03). Patients with GD were diagnosed with cancer at a median (range) of 0.5 years (1 month to 7 years) before the index date, whereas for controls, the median time was 1.8 years (10 months to 6 years) (*p* = 0.1). Six cases were diagnosed within the six months prior to the index date.

### 3.2. Cancer Incidence after Index Date in Patients with GD Compared to Controls

Cancer was diagnosed in 57 individuals after the index date, 20 (7.9%, 95% CI 4.9–11.9%) among patients with GD and 37 (1.1%, 95% CI 0.8–1.5%) among controls (Table 3). The commonest types of cancers in patients with GD were hematological, brain, colon, and NMSC.

**Figure 1:**
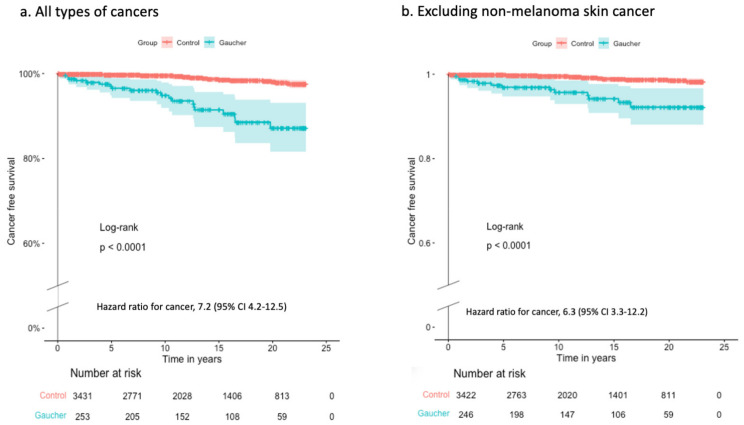
Cancer-free survival during follow-up after the index date for (**a**) all types of cancer and (**b**) excluding non-melanoma skin cancer. Subjects with a cancer diagnosis prior to the index date or no follow-up data were excluded. From the cohort (**b**), subjects with a diagnosis of non-melanoma skin cancer were also excluded. Comparison between patients and controls was conducted using the log-rank test, and the hazard ratio with a 95% confidence interval was calculated.

Patients with GD were diagnosed with cancer at a median (range) of 9.4 years (2 months to 19.8 years) after the index date, whereas for controls, the median was 12.2 years (1 month to 21.6 years) (*p* = 0.08). Two cases, one patient and one control, were diagnosed during the six months after the index date. Patients with GD had a significantly higher risk of developing cancer than controls over a comparable follow-up time (Figure 1).

The median (range) age at cancer diagnosis was 62 (36–79) and 46 (26.5–91) years in patients with GD and controls, respectively (*p* = 0.02). The incidence of cancer (excluding NMSC) was 4.14 (95% CI 2.2–7.08) per 1000 patient-years in patients with GD compared to 0.65 (95% CI 0.43–0.94) per 1000 patient-years in controls, resulting in an incidence rate ratio of 6.37 (95% CI 3.03–12.72).

Four patients with GD (1.5%) were diagnosed with more than one type of cancer after the index date; two patients had two types of NMSC, one patient had colon cancer, NMSC, and non-Hodgkin lymphoma, and one patient had melanoma, NMSC, prostate, and lung cancer. None of the controls had multiple primary cancers.

### 3.3. Cancer-Associated Risk Factors and Screening Tests in Patients with GD Compared to Controls

No significant differences were found between patients with GD and controls in the prevalence of ICD9 codes for family history of cancer (2.3% vs. 3.2%), smoking (past/current) (1.5% in both groups), diabetes mellitus (5.3% vs. 5%), or use of contraceptives (12.9% vs. 13.4%).

Before the index date, no significant differences were found between patients with GD and controls in the rate of cancer screening tests performed at least once; colonoscopy was performed in 5.3% vs. 3%, occult blood in stool in 7.2% vs. 6.5%, mammography in 13.4% vs. 10.1% of women, PAP smear in 9% vs. 10.2% of women, PSA test in 11.5% vs. 12% of men, and skin screen in 0% in both patients with GD and controls.

After the index date, at least one colonoscopy and skin screens were performed significantly more frequently in patients with GD compared to controls (Table 4). Slightly higher rates of PAP smear and PSA tests were performed in patients with GD. The frequency of occult blood testing and mammogram performance did not differ between the groups.

### 3.4. Risk Factors for Cancer in Patients with GD

In this sub-analysis of patients older than 20 years with at least one year of follow-up, we included 176 patients with GD, 18 (10.2%) of whom developed cancer after the index date (Table 5). In the univariate analysis, patients who developed cancer were older at the time of the index date and less likely to be treated with GD-specific medication compared to those who remained cancer-free (Table 5). In the multivariate analysis, only age at the index date was found to be associated with cancer risk. A significant age difference was found between patients who received treatment for GD and those who did not; treated patients had a median age of 34 (range 20 to 88) years, while untreated patients had a median age of 42 (range 22 to 77) years.

## 4. Discussion

This retrospective observational analysis used real-world data to assess the risk of cancer diagnosis in patients with GD and controls. We found that patients with GD had a higher risk of cancer than controls before and after the index date for the diagnosis of GD. The main types of cancers diagnosed in patients with GD were hematological, NMSC, colon, and brain tumors.

The primary significance of this matched comparative study between patients with GD and controls is that it addresses the ascertainment bias present in previous reports, where cancer rates were compared to non-matched cohorts. The age-standardized incidence rates (ASRs) for all cancers and specific cancer sites differ between populations; the male and female ASR was higher in the United States (US) Surveillance, Epidemiology and End Results (SEER) data than in the Israel National Cancer Registry (INCR) [12]. Thus, studies that compared the Gaucher cohort, with a high percentage of Ashkenazi Jewish individuals, to the US SEER data may be misleading [8,10]. In fact, in a study from a US Gaucher clinic, the relative risk for cancer in GD was lower when compared to US SEER data, 1.80 (1.32–2.40), than when compared to the INCR data, 2.45 (1.79–3.27) [5]. The higher risk for breast cancer in the ICCG study [10] may have been biased, as both the occurrence of GD and risk of breast cancer are greater for women of Ashkenazi Jewish heritage.

The second significant implication was our ability to study the associations with variables associated with higher cancer risk, such as a family history of cancer, smoking, and the performance of cancer screening tests. The presence of ICD-9 codes for family cancer history and smoking was found to be similar between patients and controls. Although smoking was recorded less frequently than expected, we can assume that there was non-differential misclassification between patients and controls.

Patients under surveillance for chronic diseases tend to undergo a higher frequency of screening tests to detect early signs of cancer. This was also observed in our cohort, where patients with GD underwent more screening tests for cancer compared to controls. A higher frequency of screening tests can lead to a higher probability of detecting cancers, and this higher probability can be misinterpreted as a higher disease risk [13]. Undergoing more skin screen tests probably increased the likelihood of being diagnosed with skin cancer, mainly NMSC [14]. More colonoscopies may have resulted in the diagnosis of early-stage cancers, increasing the incidence of colon cancer [15].

Early cancer diagnosis can also be related to the routine evaluations performed at GD follow-up. Even without specific cancer screening, undergoing routine tests for GD, such as a complete blood count, may lead to early detection of colon cancer by showing a reduction in hemoglobin levels, even within the normal range [16]. Abdominal imaging performed to evaluate spleen and liver volume may lead to the detection of early-onset abdominal malignancies. Following the immunoglobulin profile regularly, as recommended [17], can potentially lead to the early detection of multiple myeloma [18].

As expected, the risk of cancer diagnoses in patients with GD increased with age [19,20]. In different cohorts, the cancer rate in patients with GD ranged from 4% to 12% depending on the median age of the study cohorts; the lowest rate of cancer diagnoses was when the median age was 30–40 years [6,8], and the highest rate was when the median age was around 48 years [4,10]. We found an 8% cancer rate in a cohort with a median age of 42 years. It was suggested that a longer time is needed for GD to induce cancer [21]. Although men have a higher risk for cancer [12], we did not find this association in our cohort, most probably because of the younger age of our cohort.

Over the years, controversy has arisen regarding the role of GD-specific therapy, e.g., enzyme replacement therapy, on cancer risk [7]. Some have shown that most cancer diagnoses occurred in untreated patients [5] or patients with treatment delays [20], while others suggested that initiating GD-specific therapy may increase cancer risk [6]. In our data, although more cancer cases occurred in untreated patients, this finding was related to the age of those patients, as untreated patients were older than treated ones. In the multivariate analysis, treatment status was not associated with cancer risk.

Multiple primary cancers were reported in 1–2.2% of GD cases [4,22,23]. Similarly, we had four cases with more than one type of cancer in the GD group, with no such case in the control group. Splenectomy, which is associated with cancer risk [24], is also associated with multiple primary cancers [20,22]. The lack of association with splenectomy in our cohort may be related to the under-coding of splenectomy in the electronic coding system.

Our study confirmed findings from previous studies that hematological malignancies are more common in patients with GD [4,5,8,10,21,25,26]. Bone marrow biopsy performed for the evaluation of suspected hematological malignancy can detect Gaucher cells and lead to the diagnosis of GD, explaining the observed increase in hematological malignancies diagnosis around the time of GD diagnosis [27]. Several hypotheses have been suggested to account for the augmented B-cell proliferation in GD, such as sphingosine derived from extra lysosomal glucosylceramide metabolism, gene modifiers, immune system dysregulation triggered by cytokines, chemokines, and chronic metabolic inflammation, and endoplasmic reticulum stress [23,25,28,29,30].

The main limitation of this study is the relatively small number of cancer cases and the young age of the study cohort, specifically as we know that the risk of cancer increases with age. Based on the lower-than-expected cancer cases compared to the INCR, we can assume missing documentation in the MHS database, which is probably non-differential between patients and controls. A further limitation is the inherent bias of controls having to be alive at the index date. Therefore, from a population perspective, fatal cancer cases that occurred prior to the index date would not be discernable.

## 5. Conclusions

The increased cancer occurrence in patients with GD can be attributed partly to a more rigorous surveillance approach and frequent screening. However, the higher rates of cancers without early detection measures indicate that there is indeed an elevated risk of developing cancers in individuals with GD, regardless of any potential bias in identifying cases.

## Figures and Tables

**Table 1:** Study cohort.

	Patients with GD	Controls
Number	264	3440
Male	130 (49%)	1689 (49%)
Age at index date *, median (range)	31 (0–88)	31 (0–88)
Age > 60 years at index date	26 (10%)	338 (10%)
Age at the end of the follow-up, median (range)	43 (4–94)	43 (2–96)
Age > 60 years at last follow-up	60 (23%)	816 (24%)

GD, Gaucher disease. * The index date was the date of the first documentation of GD. For controls, the index date was the index date of the matched patient with GD.

**Table 2:** Cancer cases diagnosed before the index date *.

	Patients with GD	Controls	OR (95% CI)	*p*-Value
Total	11/264 (4.2%)	7/3441 (0.2%)	21 (8.2–55.5)	*p* < 0.001
Cervical	0	5	-	NS
Hematology ^	3	0	91 (4.8–1763)	<0.01
NMSC	4	0	118 (6.3–2214)	<0.01
Prostate	1	0	39 (1.6–958)	0.02
Respiratory	1	0	39 (1.6–958)	0.02
Connective tissue	1	0	39 (1.6–958)	0.02
Unspecified	1	0	39 (1.6–958)	0.02
Other	0	2	-	NS

GD, Gaucher disease; OR, odd ratio; CI, confidence interval; NS, non-significant. NMSC, non-melanoma skin cancer. * The index date was the date of the first documentation of GD. For controls, the index date was the index date of the matched patient with GD. ^ leukemia (*n* = 1), multiple myeloma (*n* = 1), non-Hodgkin lymphoma (*n* = 1).

**Table 3:** Incidence rates of cancers diagnosed after the index date *.

	Patients with GD, *n* = 253		Controls, *n* = 3431		
Cancer cases	20 (7.9%)		37 (1.1%)		
Median follow-up, years	12.8		12.9		
Patient-years (PY)	3220.9		43,237.2		
	***n***	**Rate per 1000 PY**	***n***	**Rate per 1000 PY**	**IRR (95% CI)**
Cervical	1	0.31 (0.01 to 1.73)	21	0.49 (0.3 to 0.74)	0.64 (95% CI 0.02–3.97)
NMSC	7	2.17 (0.87 to 4.48)	9	0.21 (0.20–0.56)	10.4 (95% CI 3.3–32)
Colon	2	0.62 (0.08 to 2.24)	1	0.02 (0 to 0.13)	26.8 (95% CI 2.5–298)
Brain	2	0.62 (0.08 to 2.24)	1	0.02 (0 to 0.13)	26.8 (95% CI 2.5–298)
Multiple myeloma	2	0.62 (0.08–2.24)	0	0	67.2 (95% CI 1.8–1000) ^
Melanoma	1	0.31 (0.01 to 1.73)	2	0.05 (0.01 to 0.17)	6.7 (95% CI 0.11–128)
Bladder	1	0.31 (0.01 to 1.73)	2	0.05 (0.01 to 0.17)	6.7 (95% CI 0.11–128)
Prostate	1	0.31 (0.01 to 1.73)	1	0.02 (0 to 0.13)	13.6 (95% CI 0.85–216)
Other	3	0.93 (0.02 to 2.72)	0	0	-

PY, patient-years; IRR, incidence rate ratio; NMSC, non-melanoma skin cancer. * The index date was the date of the first documentation of Gaucher disease (GD). For controls, the index date was the index date of the matched patient with GD. ^ For the IRR analysis, 0.5 was added to both groups. The IRR and 95% CI between the rate among patients with GD compared to controls were calculated.

**Table 4:** Frequency of conducting at least one screening test after the index date *.

	Patients with GD	Controls	*p*-Value
Male/Female	130/134	1689/1751	
Colonoscopy	79 (29.9%)	662 (19.2%)	<0.001
Occult blood in stool	86 (32.6%)	983 (28.6%)	0.19
Mammography ^$^	64 (47.8%)	605 (42.5%)	0.58
PAP smear ^$^	19 (14.2%)	152 (8.7%)	0.048
PSA test ^	55 (42.3%)	554 (32.5%)	0.024
Skin screen	20 (7.6%)	86 (2.5%)	<0.001

GD, Gaucher disease; PAP, Papanicolaou; PSA, prostate-specific antigen. * The index date was the date of the first documentation of GD. For controls, the index date was the index date of the matched patient with GD. ^$^ Calculated from the female cohort. ^ Calculated from the male cohort.

**Table 5:** Risk factors associated with cancer diagnosis in patients with Gaucher disease.

	Cancer *n* = 18	No Cancer *n* = 158	Univariate, HR (95% CI)	*p*-Value	Multivariate, HR (95% CI)	*p*-Value
Age at index date, years *	52 (22–72)	35.5 (20–88)	1.06 (1.03–1.1)	0.0004	1.06 (1.02–1.1)	0.002
Male	17 (54.8%)	8 (18.2%)	0.79 (0.3–2.06)	0.62	0.68 (0.26–1.8)	0.4
SES status *	6.5 (3–9)	7 (1–10)	0.89 (0.74–1.08)	0.24	0.92 (0.75–1.12)	0.4
Splenectomy	1 (5.6%)	6 (3.8%)	2.27 (0.29–17.2)	0.43	1.35 (0.17–10.3)	0.77
GD medication	6 (33%)	93 (58.8%)	0.36 (0.13–0.96)	0.04	0.42 (0.15–1.15)	0.09

* Median (range). HR, hazard ratio; CI, confidence interval; SES, socioeconomic; GD, Gaucher disease. Univariate and multivariate Cox proportional hazard analysis was used for 176 patients older than 20 years with a follow-up of at least one year.

## Data Availability

The data presented in this study are available on request from the corresponding author.
